# *Clinacanthus nutans* attenuates atherosclerosis progression in rats with type 2 diabetes by reducing vascular oxidative stress and inflammation

**DOI:** 10.1080/13880209.2021.1990357

**Published:** 2021-10-25

**Authors:** Ahmad Khusairi Azemi, Siti Safiah Mokhtar, Sharifah Emilia Tuan Sharif, Aida Hanum Ghulam Rasool

**Affiliations:** a Department of Pharmacology, School of Medical Sciences, Universiti Sains Malaysia, Kota Bharu, Kelantan, Malaysia; b Department of Pathology, School of Medical Sciences, Universiti Sains Malaysia, Kota Bharu, Kelantan, Malaysia; c Hospital Universiti Sains Malaysia, Kota Bharu, Kelantan, Kota Bharu, Malaysia

**Keywords:** SOD, MDA, intima-media thickness, atherogenic index, TNF-α, anti-atherosclerosis

## Abstract

**Context:**

Atherosclerosis predisposes individuals to adverse cardiovascular events. *Clinacanthus nutans* L. (Acanthaceae) is a traditional remedy used for diabetes and inflammatory conditions.

**Objectives:**

To investigate the anti-atherosclerotic activity of a *C. nutans* leaf methanol extract (CNME) in a type 2 diabetic (T2D) rat model induced by a high-fat diet (HFD) and low-dose streptozotocin.

**Materials and methods:**

Sixty male Sprague-Dawley rats were divided into five groups: non-diabetic fed a standard diet (C), C + CNME (500 mg/kg, orally), diabetic fed an HFD (DM), DM + CNME (500 mg/kg), and DM + Metformin (DM + Met; 300 mg/kg). Treatment with oral CNME and metformin was administered for 4 weeks. Fasting blood glucose (FBG), serum lipid profile, atherogenic index (AI), aortic tissue superoxide dismutase levels (SOD), malondialdehyde (MDA), and tumour necrosis factor-alpha (TNF-α) were measured. The rats’ aortas were stained for histological analysis and intima-media thickness (IMT), a marker of subclinical atherosclerosis.

**Results:**

The CNME-treated diabetic rats had reduced serum total cholesterol (43.74%; *p* = 0.0031), triglycerides (80.91%; *p* = 0.0003), low-density lipoprotein cholesterol (56.64%; *p* = 0.0008), AI (51.32%; *p* < 0.0001), MDA (60.74%; *p* = 0.0026), TNF-α (61.78%; *p* = 0.0002), and IMT (39.35%; *p* < 0.0001) compared to untreated diabetic rats. SOD level, however, increased (53.36%; *p* = 0.0326). These CNME effects were comparable to those in the metformin-treated diabetic rats.

**Conclusions:**

*C. nutans* possesses anti-atherosclerotic properties, which may be due to reductions in vascular tissue oxidative stress, inflammation, and serum AI. Continued studies on atherosclerotic animal models are suggested.

## Introduction

Diabetes mellitus is a known risk factor for atherosclerosis and cardiovascular diseases. The prevalence of diabetes mellitus has increased globally in recent years. According to the International Diabetes Federation (International Diabetes Federation 2019), approximately 463 million adults (20–79 years) were living with diabetes. This figure is predicted to rise to 700 million by the year 2045. The proportion of people with type 2 diabetes (T2D) especially is increasing in most countries (International Diabetes Federation 2019). The high prevalence of diabetes is expected to contribute to the increased prevalence of cardiovascular diseases related to atherosclerosis, including stroke, coronary artery disease, and peripheral vascular disease (Wihastuti et al. 2018). Atherosclerosis refers to the progressive thickening and hardening of the walls of medium and large arteries as a result of fat deposits on their inner lining. It is a multi-faceted disease accompanied by endothelial dysfunction, oxidative stress, inflammation, foam cell formation, plaque rupture, and occluding thrombus (Nelson et al. 2017).

Studies have observed that disorders induced by high-fat diets (HFD) resemble human metabolic syndrome, with implications for cardiovascular health. Obesity and dyslipidemia are components of metabolic syndrome, which is commonly seen in T2D. Dyslipidemia is associated with the altered physical properties of cellular membranes, which may facilitate the escape of free radicals from the mitochondrial electron transport chain or activate nicotinamide adenine dinucleotide phosphate oxidase (Sharma et al. 2012). These cause increased free radical species generation and increases their tendency to react with other molecules. This results in the oxidation and peroxidation of lipids, proteins, and lipoproteins (Abbasnezhad et al. 2019). Peroxidation of the endothelial cell membrane can lead to endothelial damage and dysfunction. Low-density lipoprotein cholesterol (LDL-C) and its oxidized form (oxLDL-C) play a significant role in atherogenesis. Increased oxLDL-C triggers tumour necrosis factor-alpha (TNF-α) gene expression, especially in vascular tissue (Niemann-Jönsson et al. 2000). Augmented TNF-α levels increase vascular permeability to blood macromolecules and cause inflammatory cells to enter the injured vascular tissue, thus contributing to the formation of atherosclerotic plaque.

Certain medicinal plants and natural products have hypoglycemic and hypolipidemic properties with fewer side effects, easy and local sourcing, and lower costs compared to conventional drugs. They may be further investigated and developed as drugs or used as an adjunct to current drugs. It is possible that some of these medicinal plants may be able to reduce diabetic vascular complications as well. One of these medicinal plants that have gained recent attention is *Clinacanthus nutans* (Burm. f) Lindau (Acanthaceae), locally known in Malaysia as Sabah snake grass. Traditionally, *C. nutans* leaves have been consumed to treat diabetes (Alam et al. 2016). In Malaysia, Thailand, and Indonesia, the plant is used externally for the treatment of snake and insect bites, as well as skin rashes (Alam et al. 2016). Scientifically, *C. nutans* extracts have been shown to exert hypoglycemic (Umar Imam et al. 2019; Azemi, Mokhtar, Rasool 2020), hypolipidemic (Sarega et al. 2016; Umar Imam et al. 2019), antioxidant (Sarega et al. 2016), and anti-inflammatory properties (Mai et al. 2016). The presence of active compounds in *C. nutans* extracts, such as protocatechuic acid (Sarega et al. 2016), caffeic acid, chlorogenic acid (Sarega et al. 2016), stigmasterols (Azemi, Mokhtar, Rasool 2020), β-sitosterols (Azemi, Mokhtar, Rasool 2020), squalene (Gabás-Rivera et al. 2014), and 5-hydroxylmethylfurfural (Azemi, Mokhtar, Rasool 2020), have been associated with these pharmacological properties.

The hypoglycemic, hypolipidemic, antioxidant, and anti-inflammatory properties of *C. nutans* extracts might contribute to preventing or reducing the severity of diabetic vasculopathy (atherosclerosis). Therefore, the present study investigates the anti-atherosclerotic activity of a *C. nutans* leaf methanol extract (CNME) on serum lipid profiles, atherogenic index (AI), vascular tissue oxidative stress, inflammation, and vascular structural changes in HFD-fed T2D rat models.

## Materials and methods

### Plant material and extraction

*C. nutans* leaves were collected from Pasir Puteh, an area in the state of Kelantan, Malaysia, between 3 January and 1 February 2017. The plant was identified and authenticated by Ms. Ummu Hani Badron, a botanist at the herbarium of Forest Research Institute Malaysia, and a voucher specimen (SBID 039/18) was deposited in the herbarium for future reference. The leaf extract was prepared according to the methods previously described by Abdul Rahim et al. (2016). The *C. nutans* leaves were washed and allowed to dry in an oven at 40 °C for 3 d, then ground into a powder using an electric grinder. The powdered leaves (125 g) were soaked in methanol (Fisher Scientific, Loughborough, England) at a ratio of 1:20 (w/v) for 72 h at room temperature. The mixture was then filtered with Whatman No.1 filter paper (Merck, Germany), and the filtrate was allowed to evaporate in a vacuum rotary evaporator (Heidolph, Germany) set at 40 °C. After the solvent was completely removed, the yield achieved was stored in vials at 4 °C until use.

### Phytochemical CNME screening

The CNME extract was subjected to phytochemical screening according to the standard conventional protocols described in previous studies (Abdul Rahim et al. 2016; Ismail Suhaimy et al. 2017).

### Alkaloid test

The CNME sample was macerated in chloroform, followed by the addition of ammoniacal chloroform. The mixture was then treated with 10% sulphuric acid and tested with Mayer’s reagent. The formation of precipitates indicated the presence of alkaloids.

### Saponin test

The methanol CNME extract was mixed with distilled water in a test tube. The formation of a stable froth for at least 15 min indicated the presence of saponins.

### Flavonoid test

The chloroform CNME extract was dissolved in ether and shaken in a 10% ammonia solution. The formation of yellow colour in the ammonia layer indicated the presence of flavonoids.

### Tannin test

The methanol CNME extract was mixed with a 1% ferric chloride solution. The formation of a blue-black colour indicated the presence of hydrolyzable tannins, while a brownish-green colour indicated that of condensed tannins.

### Triterpene and phytosterol test

The chloroform CNME extract was tested using a Lieberman–Buchard reagent. The formation of a reddish colour indicated the presence of triterpenes, and that of a greenish colour signalled the presence of phytosterols.

### HFD preparation

An HFD was prepared according to the methods outlined by Ishak et al. (2013) and Lim et al. (2016) with some modifications. The HFD was prepared from a mixture of 50% standard rat chow (Gold Coin Feedmills, Port Klang, Malaysia), 38% ghee, 8% full cream milk powder, and 4% white sugar.

### Animals

The Universiti Sains Malaysia (USM) Institutional Animal Care and Use Committee (USM IACUC) [USM/IACUC/2017/(105)(834)] granted ethical approval for the study. Sixty male Sprague-Dawley rats (age: 12 weeks; weight: 250–300 g) were used for the study. They were acclimatized for 1 week in a temperature-controlled room (22 °C) with a 12 h light/dark cycle, and fed standard rat chow and water ad libitum. After acclimatizing, the rats were divided into non-diabetic and diabetic groups. The rats in the non-diabetic groups were fed standard rat chow, while the diabetic groups were fed the HFD. After 4 weeks on their respective diets, the rats in the diabetic groups were injected intraperitoneally with a single dose of streptozotocin (40 mg/kg, dissolved in 10 mM citrate buffer, pH 4.5, 1 mL/kg). The rats in the non-diabetic groups were injected with an equal volume of citrate buffer (1 mL/kg, intraperitoneally; Ishak et al. 2013; Mokhtar et al. 2016). After 1 week of diabetes induction, hyperglycemia was confirmed in the rats by measuring the concentration of fasting blood glucose (FBG) from the tail tip using a one-touch glucometer (Accu-check, Roche Diagnostic, Indianapolis, IN, USA). The rats with an FBG concentration ≥ 16.7 mmol/L were considered diabetic (Oztürk et al. 2015; Jia et al. 2017). After that, all rats continued with their respective diets until week 11, when the non-diabetic and diabetic rats were divided into five groups (*n* = 12):

Group 1: Non-diabetic control rats (C)
Group 2: Non-diabetic rats treated with 500 mg/kg daily of CNME extract (C + CNME)
Group 3: Untreated diabetic rats (DM)
Group 4: Diabetic rats treated with 300 mg/kg daily of metformin (DM + Met)
Group 5: Diabetic rats treated with 500 mg/kg daily of CNME extract (DM + CNME)

The treatments were given via oral gavage for 4 weeks. All rats were sacrificed using a combination of ketamine and xylazine (300:30 mg/kg). Blood samples were collected from a renal vein and used for lipid profile analyses. Thoracic aortas were dissected out for biochemical and histology analyses.

### Serum lipid profile and AI measurement

The blood samples were centrifuged at 1500 *g* (Kubota 4000, Japan) for 25 min, and the resulting serum was used for lipid profile analyses. Serum total cholesterol (TC), triglycerides (TG), and high-density lipoprotein cholesterol (HDL-C) were measured using the colorimetric method with an Integra 800 automatic immunoanalyzer (Roche Diagnostic Systems, Mannheim, Germany). The serum LDL-C was calculated using the Friedwald formula (Oztürk et al. 2015), as follows:

LDL−C=TC−(HDL−C + TG/5)

The AI is a strong marker for predicting the risk of atherosclerosis and coronary heart disease (Kazemi et al. 2018). Its formula is based on the LDL-C and HDL-C values in the serum (Jiang et al. 2017), calculated as follows:

AI=LDL−C/HDL−C

### Biochemical analyses of thoracic aorta tissue lysate

#### Protein concentration measurement

The thoracic aortas were isolated from the rats and homogenized in a lysis buffer (radioimmunoprecipitation assay buffer, Sigma Chemical Co., St Louis, MO, USA) containing a 0.05% protease inhibitor cocktail (Sigma Chemical Co., St Louis, MO, USA). After centrifugation at 3000 *g* for 20 min at 4 °C, the supernatants were collected. Protein concentrations were determined using a protein determination kit (Cayman Chemicals, USA). The supernatants were used for analyses of superoxide dismutase (SOD), malondialdehyde (MDA), and TNF-α levels.

#### Aortic SOD activity and MDA measurement

Aortic tissue SOD activity was measured using an EnzyChrome SOD assay kit (Catalog number ESOD100) purchased from BioAssay Systems (Hayward, CA, USA) and performed according to the procedures detailed in the kit. SOD activity was expressed as U/mg protein (Zhu et al. 2011). Aortic tissue MDA levels were measured using an NWLSS^TM^ MDA assay kit (catalog number NWK-MDA01) purchased from Northwest Life Science Specialties, LLC (Vancouver, USA) and performed according to the detailed procedures in the kit. MDA levels were expressed as nmol/mg protein (Zhu et al. 2011).

#### Aortic TNF-α measurement

Aortic tissue TNF-α levels were measured using an enzyme-linked immunosorbent assay kit (catalog number KRC 3011) according to the manufacturer’s detailed instructions (ThermoFisher Scientific, Vienna, Austria).

#### Histology

The histology study was carried out according to the method described by Azemi, Mokhtar, Low, et al. (2020). The thoracic aortas were preserved in 10% formalin. The tissues were then dehydrated using graded ethanol, embedded in paraffin blocks, and cut into 4 µm thick sections to be mounted on glass slides. The sections were stained with haematoxylin and eosin and then viewed under a light microscope at a magnification of ×400 (Olympus BX41, Olympus America Inc., Centre Valley, PA, USA). Images were captured via microscope camera (Olympus XC50, Olympus America Inc., Centre Valley, PA, USA), and the sections were then analyzed using cellSens imaging software (Olympus America Inc., Centre Valley, PA, USA). Four different parts per image of the aortic circumference were obtained at 0°, 90°, 180°, and 270° to estimate the intima-media thickness (IMT).

### Statistical analyses

Statistical analyses were carried out using GraphPad Prism software (v.7.0; San Diego, CA, USA). Group comparisons were assessed via one-way analysis of variance, followed by Bonferroni’s test. Values were expressed as mean ± standard error of the mean (SEM). *p*-Values less than 0.05 were considered statistically significant.

## Results

### Phytochemical CNME screening

The phytochemical CNME screening showed the presence of flavonoids, saponins, tannins, and phytosterols in the extract.

### General characteristics

**Figure 1.**
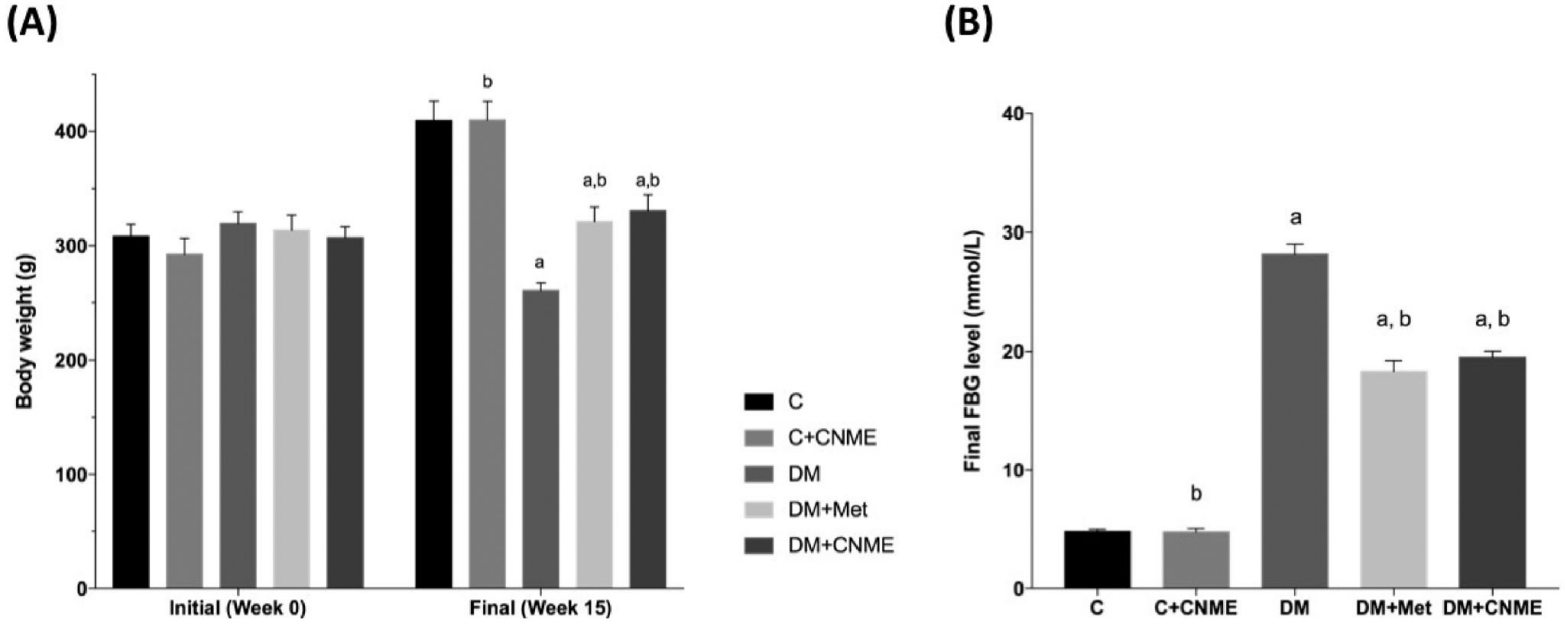
The study groups’ (A) body weights and (B) fasting blood glucose (FBG) levels at the end of 15 weeks. The diabetic groups treated with CNME or metformin increased in body weight and had reduced FBG levels compared to the untreated diabetic group. Data are presented as mean ± SEM (*n* = 12). ^a^*p* < 0.05 vs. C group. ^b^*p* < 0.05 vs. DM group. Non-diabetic control group: C; non-diabetic group treated with 500 mg/kg CNME: C + CNME; untreated diabetic group: DM; diabetic group treated with 300 mg/kg metformin: DM + Met; diabetic group treated with 500 mg/kg CNME: DM + CNME.

The body weights of the rats in the five study groups are shown in Figure 1(A). There was no difference in initial body weight among the study groups. The rats in groups DM + Met and DM + CNME gained significantly more weight at the end of the study period compared to those in the untreated DM group (*p* < 0.05; Figure 1(A)). The final FBG levels in the untreated and diabetic groups treated with CNME and metformin were higher than the non-diabetic control groups (*p* < 0.05). However, both the DM + Met and DM + CNME groups had reduced FBG levels compared to the DM group (*p* < 0.05; Figure 1(B)).

### Serum TC, TG, HDL-C, LDL-C, and AI levels

Compared to those in the C group, the rats in the DM group showed higher serum TC (DM: 5.67 ± 0.77 vs. C: 1.85 ± 0.10 mmol/L; *p* < 0.0001), TG (DM: 14.77 ± 3.62 vs. C: 0.63 ± 0.10 mmol/L; *p* < 0.0001), LDL-C (DM: 2.26 ± 0.47 vs. C: 0.86 ± 0.09 mmol/L; *p* < 0.001), and AI (DM: 1.89 ± 0.19 vs. C: 0.87 ± 0.10; *p* < 0.001). The diabetic rats in the DM + CNME (*p* < 0.05) and DM + Met groups (*p* < 0.05) also showed lower serum TC, TG, LDL-C, and AI levels compared to rats in the DM group (Table 1). No significant difference emerged between the C + CNME and C groups.

**Table 1.** Levels of TC, TG, HDL-C, LDL-C and atherogenic index in serum of rats at week 15.

Group	TC (mmol/L)	TG (mmol/L)	HDL-C (mmol/L)	LDL-C (mmol/L)	AI (LDL-C/HDL-C)
C	1.85 ± 0.10	0.63 ± 0.10	0.97 ± 0.17	0.86 ± 0.09	0.87 ± 0.10
C + CNME	1.92 ± 0.11****	0.69 ± 0.19****	1.07 ± 0.26	0.86 ± 0.11**	0.89 ± 0.11**
DM	5.67 ± 0.77^####^	14.77 ± 3.62^####^	1.20 ± 0.07	2.26 ± 0.47^###^	1.89 ± 0.19^###^
DM + Met	3.19 ± 0.40**	3.49 ± 0.70***	1.23 ± 0.08	0.69 ± 0.08****	0.61 ± 0.08****
DM + CNME	3.19 ± 0.45**	2.82 ± 0.84***	1.18 ± 0.05	0.98 ± 0.13***	0.92 ± 0.22**

Values are expressed as mean ± SEM (*n* = 12). ^###^*p* < 0.001, ^####^*p* < 0.0001 versus C group. **p < 0.01, ***p < 0.001, ****p < 0.0001 vs. DM group. Non-diabetic control group: C; non-diabetic group treated with 500 mg/kg CNME: C + CNME; untreated diabetic group: DM; diabetic group treated with 300 mg/kg metformin: DM + Met; diabetic group treated with 500 mg/kg CNME: DM + CNME.

### Aortic tissue MDA level and SOD activity

**Figure 2.**
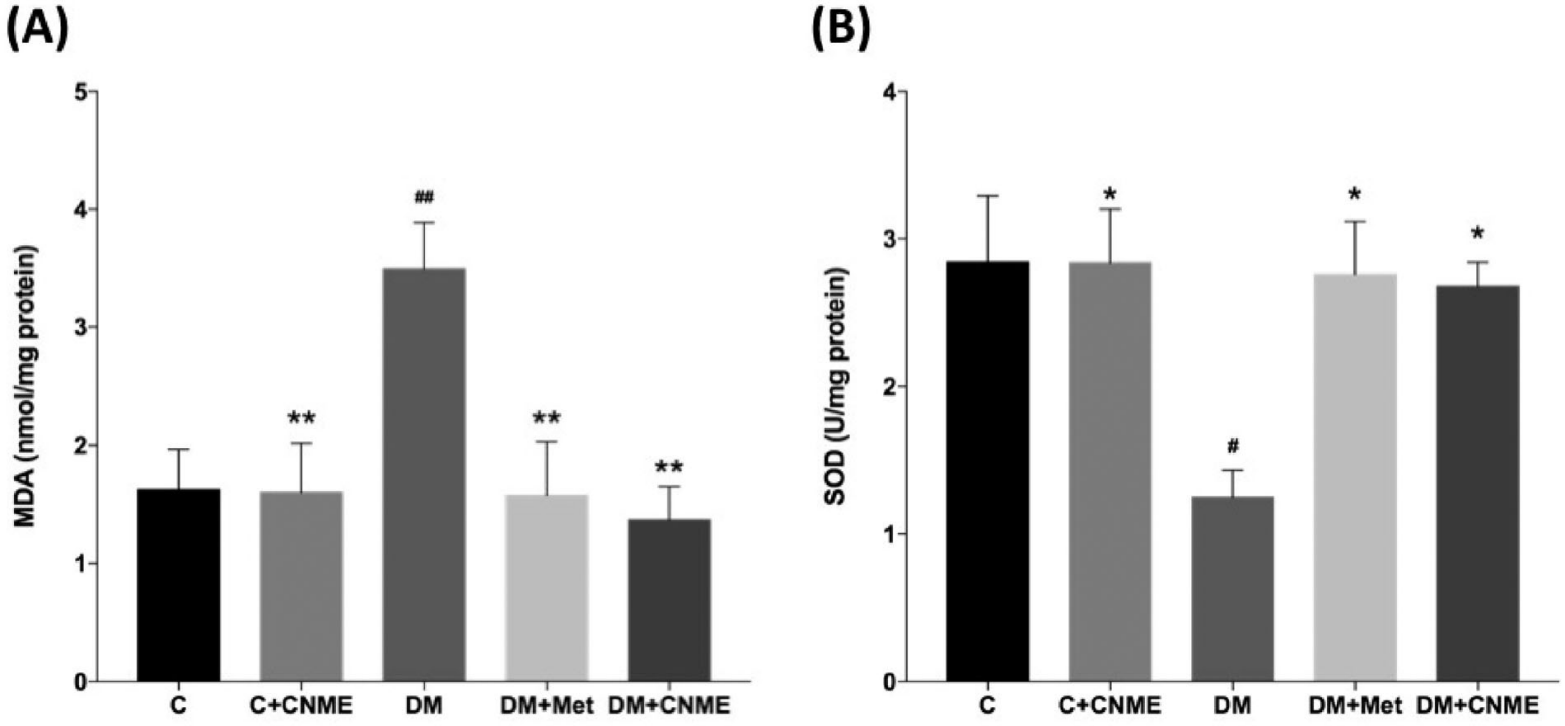
(A) MDA and (B) SOD levels in the thoracic aortas of non-diabetic and diabetic rats. The diabetic groups treated with CNME or metformin had reduced MDA and increased SOD activity compared to the untreated diabetic group. Data are presented as mean ± SEM (*n* = 12). ^#^*p* < 0.05, ^##^*p* < 0.01 vs. C group. *p < 0.05, **p < 0.01 vs. DM group. Non-diabetic control group: C; non-diabetic group treated with 500 mg/kg CNME: C + CNME; untreated diabetic group: DM; diabetic group treated with 300 mg/kg metformin: DM + Met; diabetic group treated with 500 mg/kg CNME: DM + CNME.

Rats in the DM group showed higher MDA (DM: 3.49 ± 0.39 vs. C: 1.62 ± 0.34 nmol/mg protein; *p* = 0.0081) levels and lower SOD activity (DM: 1.25 ± 0.18 vs. C: 2.84 ± 0.25 U/mg protein; *p* = 0.0117) compared with those in the C group (Figure 2(A,B), respectively). No significant difference occurred between the C + CNME and C groups. The C + CNME group also had lower MDA levels (DM: 3.49 ± 0.39 vs. C + CNME: 1.60 ± 0.41 nmol/mg protein; *p* = 0.0096) and higher SOD activity (DM: 1.25 ± 0.18 vs. C + CNME: 2.84 ± 0.36 U/mg protein; *p* = 0.0156) compared to the untreated DM group. Both the DM + CNME and DM + Met groups had significantly reduced MDA levels (DM: 3.49 ± 0.39 vs. DM + CNME: 1.37 ± 0.28 nmol/mg protein, *p* = 0.0026; DM: 3.49 ± 0.39 vs. DM + Met: 1.58 ± 0.46 nmol/mg protein, *p* = 0.0083) and increased SOD activity (DM: 1.25 ± 0.18 vs. DM + CNME: 2.68 ± 0.16 U/mg protein, *p* = 0.0326; DM 1.25 ± 0.18 vs. DM + Met: 2.76 ± 0.36 U/mg protein, *p* = 0.0249) compared to the DM group (Figure 2(A,B), respectively).

### Aortic tissue TNF-α level

**Figure 3.**
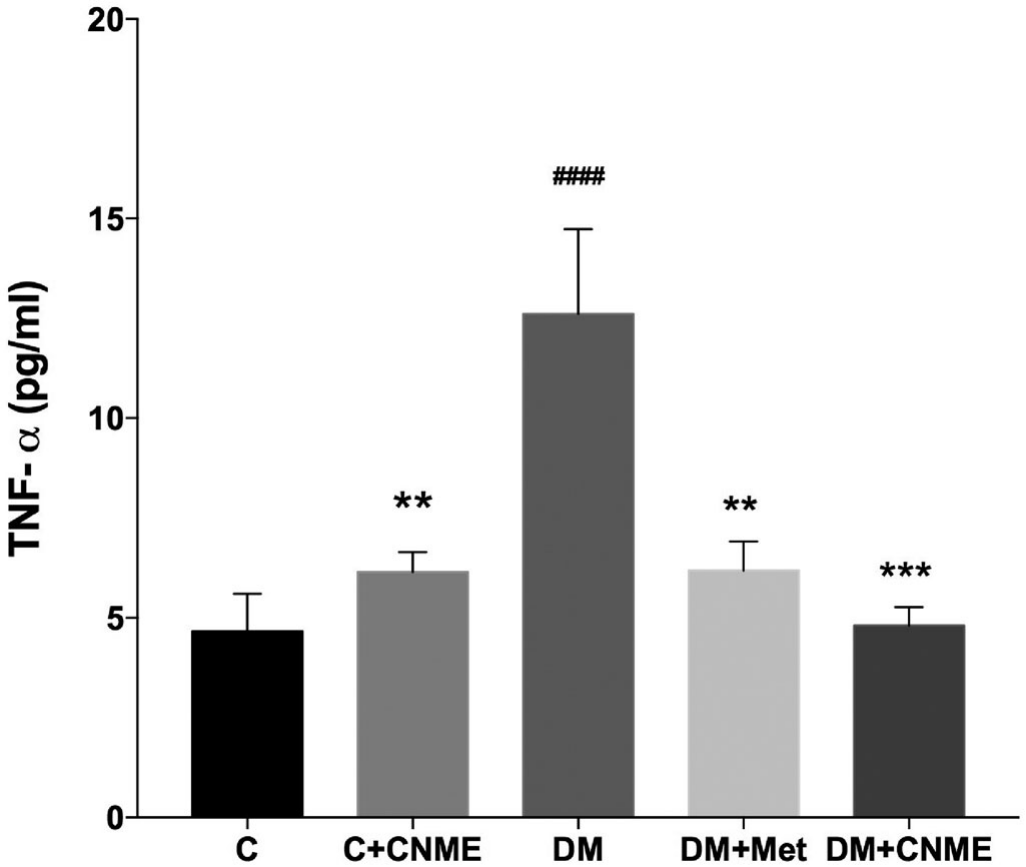
TNF-α levels in the study group’s thoracic aortas. The diabetic groups treated with CNME or metformin had reduced vascular TNF-α levels compared to the untreated diabetic group. Data are presented as mean ± SEM (*n* = 12). ^####^*p* < 0.0001 vs. C group. **p < 0.01, ***p < 0.001 vs. DM group. Non-diabetic control group: C; non-diabetic group treated with 500 mg/kg CNME: C + CNME; untreated diabetic group: DM; diabetic group treated with 300 mg/kg metformin: DM + Met; diabetic group treated with 500 mg/kg CNME: DM + CNME.

Compared to the C group, the level of the pro-inflammatory cytokine TNF-α (DM: 12.61 ± 2.12 vs. C: 4.67 ± 0.94 pg/mL; *p* < 0.0001) was significantly higher in the aortas of the DM group (Figure 3). The C + CNME group similarly showed lower TNF-α levels (DM: 12.61 ± 2.12 vs. C + CNME: 6.16 ± 0.49 pg/mL; *p* = 0.0023) compared to the untreated diabetic rats. Likewise, compared to the untreated DM group, both diabetic groups treated with CNME (DM: 12.61 ± 2.12 vs. DM + CNME: 4.82 ± 0.46 pg/mL; *p* = 0.0002) and metformin (DM: 12.61 ± 2.12 vs. DM + Met: 6.19 ± 0.73 pg/mL; *p* = 0.0018) had significantly reduced TNF-α levels (Figure 3).

### Histological changes in the thoracic aorta

**Figure 4.**
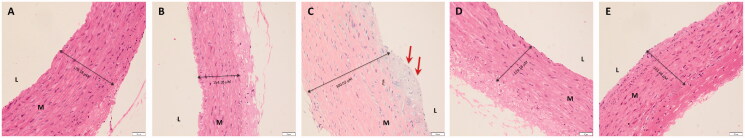
Histopathological changes in the rats’ thoracic aortas after 4 weeks of CNME treatment (magnification, ×400). (A) The non-diabetic control group (C) showed an intact vascular layer and no impairment of the vessel wall. (B) The non-diabetic group treated with 500 mg/kg CNME (C + CNME) showed an intact vascular layer and no impairment to vessel integrity. (C) In the untreated diabetic group (DM), the aortas appeared to be thick and exhibited disorientation of the smooth muscle cells with foam cell formation. (D) In the diabetic group treated with 300 mg/kg metformin (DM + Met), the aortas showed a thinner vascular wall compared to the untreated diabetic group’s aortas with no foam cell formation. (E) In the diabetic group treated with 500 mg/kg CNME (DM + CNME), the aortas had a thinner vascular wall compared to the untreated diabetic aortas, no impairment of the vascular wall, and no foam cell formation. The double arrow shows the IMT measurement, the red arrows indicate the foam cells, L represents the vascular lumen, and M indicates Media.

Figure 4 shows the histology image of the haematoxylin and eosin staining of the thoracic aorta. The aortas of the rats in the C and C + CNME groups showed an intact vascular layer and no impairment of the vessels’ integrity (Figure 4(A,B)). In the untreated DM group, the aortas appeared to be thick and exhibited disorientation of the smooth muscle cells, as well as foam cell formation (Figure 4(C)). Compared to the untreated DM group, the histology section of the aortas in the DM + CNME and DM + Met groups showed slightly thinner vascular walls, and there was no foam cell formation (Figure 4(D,E)).

**Figure 5.**
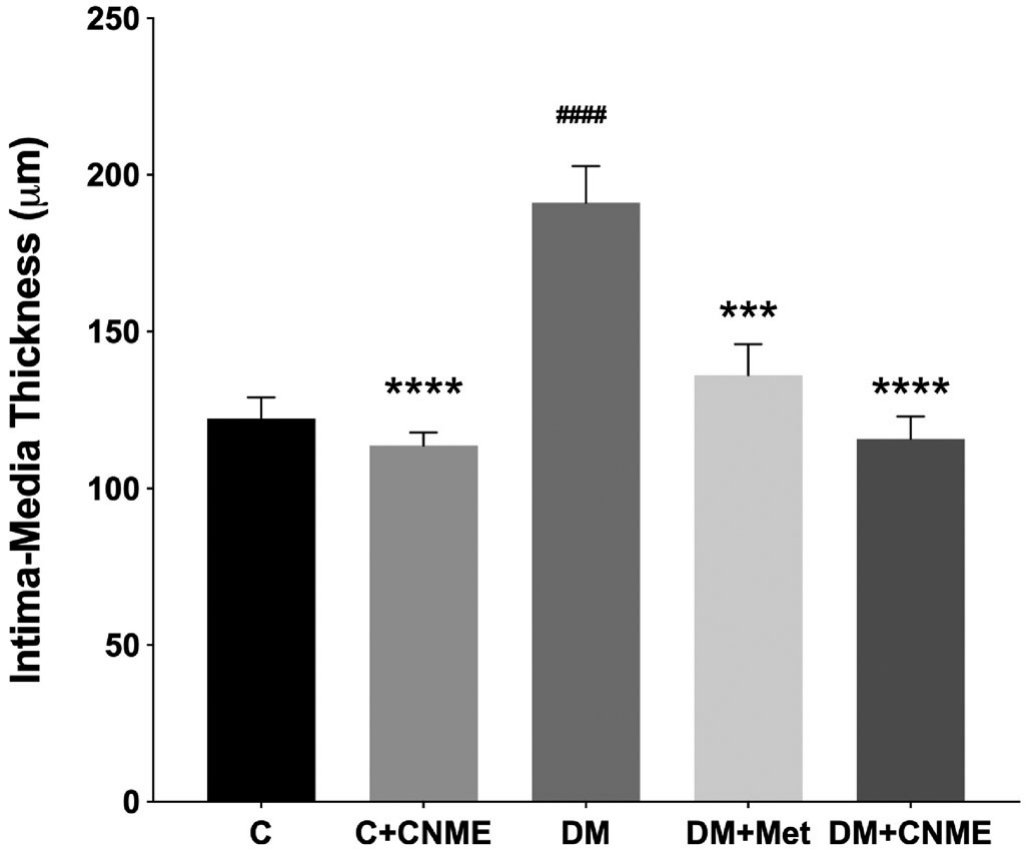
IMT of the thoracic aorta in the non-diabetic and diabetic rats treated with CNME. The diabetic groups treated with CNME or metformin had reduced aortic IMT compared to the untreated diabetic group. Data are presented as mean ± SEM (*n* = 12). ^####^*p* < 0.0001 vs. C group. ***p < 0.001, ****p < 0.0001 vs. DM group. Non-diabetic control group: C; non-diabetic group treated with 500 mg/kg CNME: C + CNME; untreated diabetic group: DM; diabetic group treated with 300 mg/kg metformin: DM + Met; diabetic group treated with 500 mg/kg CNME: DM + CNME.

Aortic IMT was examined to measure the thickness of the intima and media, the two inner layers of the thoracic aorta, after treatment with CNME and metformin. The aortic IMT in the DM group (DM: 191.10 ± 11.69 vs. C: 122.20 ± 6.72 µm; *p* < 0.0001) was 1.56 times higher compared to the C group (Figure 5). Non-diabetic rats treated with CNME also demonstrated low aortic IMT compared to the DM group (DM: 191.10 ± 11.69 vs. C + CNME: 113.70 ± 4.12 µm; *p* < 0.0001). No difference emerged between the C + CNME and C groups in their aortic IMT (Figure 5). In the DM + CNME group, IMT was likewise 1.65 times lower compared to the DM group (DM: 191.10 ± 11.69 vs. DM + CNME: 115.90 ± 7.04 µm; *p* < 0.0001; Figure 5). The rats in the DM + Met group also showed 1.41 times lower IMT than the DM group’s average (DM: 191.10 ± 11.69 vs. DM + Met: 136.00 ± 10.02 µm; *p* = 0.0002). No significant differences appeared between the DM + CNME and DM + Met groups.

## Discussion

hyperlipidemia, vascular oxidative stress, and inflammation are involved in the initiation and development of atherosclerosis (Jiang et al. 2017). In this study, T2D rats exhibited significant hyperglycemia and hyperlipidemia, as well as higher AI levels, vascular oxidative stress, and inflammation. These indices (hyperlipidemia, high AI, increased oxidative stress, and inflammation) provided a suitable model to evaluate the anti-atherosclerotic activity of CNME. This study demonstrated that CNME treatment in diabetic rats significantly reduced FBG, TC, TG, LDL-C, and AI levels compared to untreated diabetic rats. In addition, the rats’ MDA and TNF-α levels significantly reduced, though SOD activity increased in the aortic tissue of the diabetic rats treated with CNME. These rats’ aortas also showed lower IMT values compared to untreated diabetic rats. These effects of CNME were comparable with the effects of metformin in diabetic rats.

The AI is a useful marker for subclinical atherosclerosis (Cure et al. 2018; Cure and Cumhur Cure 2020). It describes the LDL-C versus HDL-C ratio, an indicator of vascular risk. This study showed untreated diabetic rats had higher AI values compared to non-diabetic rats. Previous studies have similarly demonstrated high AI in diabetic (Eleazu et al. 2014; Chikezie et al. 2020) and atherosclerotic rat models (Jiang et al. 2017; Othman et al. 2019). LDL-C undergoes oxidation (oxLDL-C) due to the increased presence of free radicals, as associated with hyperlipidemia. The accumulation of oxLDL-C in the arterial wall is an essential step in endothelial damage and atherogenesis. Therefore, reducing the level of LDL-C and inhibiting its oxidation is likely to prevent the initiation of atherosclerosis. In this study, the DM + CNME group showed significant reductions in serum TC (43.74%), TG (80.91%), and LDL-C levels (56.64%) compared to the untreated DM group. These effects of CNME were comparable with those in the metformin-treated diabetic rats. Two previous studies have similarly shown the lipid-lowering effects of *C. nutans*. Sarega et al. (2016) also demonstrated that the oral administration of aqueous methanol *C. nutans* leaf extracts at a dose of 500 mg/kg reduced TC, TG, and LDL-C by 28.04%, 32.65%, and 25.38%, respectively in a hypercholesterolemic rat model. Furthermore, Umar Imam et al. (2019) showed that oral administration of an aqueous *C. nutans* extract at a dose of 200 mg/kg in a T2D rat model reduced TC, TG, and LDL-C by 29.41%, 60.00%, and 50.00%, respectively. Additionally, Wan et al. (2013) indicated that chlorogenic acid, a phenolic compound present in *C. nutans* extract, reduced the AI level in hypercholesterolemic rats induced with a high-cholesterol diet. However, the mechanism whereby chlorogenic acid and *C. nutans* extract reduce cholesterol and AI in hypercholesterolemic rats has not been studied. Since the lipid-lowering effects of CNME and metformin are comparable in diabetic rats, it is possible that *C. nutans* has a hypolipidemic action mechanism similar to metformin as well. A previous study reported that LDL-C reduction in T2D patients treated with metformin occurs via the inhibition of intestinal bile acid reabsorption (Sonne et al. 2015). By preventing this reabsorption, bile acids are no longer transferred back to the liver. This condition leads to the increased synthesis of bile acids in the liver, which requires more cholesterol and thus reduces the amount of cholesterol in the liver (Sonne et al. 2015). Related to this, Chavez-Santoscoy et al. (2014) found that flavonoid and saponin extracts can prevent hepatic lipogenesis and stimulate cholesterol excretion via bile acid synthesis. This may be one possible mechanism for the lipid-lowering effect of *C. nutans*.

Diabetes-associated hyperglycemia causes excessive reactive oxygen species production, which can lead to an increase in inflammatory mediator expression and the subsequent progression of atherosclerosis (Zhu et al. 2011; Yuan et al. 2019). In the present study, higher MDA levels and lower SOD activities in the aortas of diabetic rats demonstrated the presence of oxidative damage and impaired antioxidant capacity. Increased aortic oxidative stress plays a significant role in the development of atherosclerosis (Azemi, Mokhtar, Low, et al. 2020). This study also demonstrated that CNME treatment reduced MDA levels, as well as increased SOD activity in the thoracic aortas of diabetic rats. The effects of CNME in reducing MDA levels and increasing SOD activity were comparable to those in metformin-treated diabetic rats. A previous study reported that the oral administration of aqueous methanolic *C. nutans* extract for 7 weeks reduced hepatic MDA levels in a hypercholesterolemic rat model (Sarega et al. 2016). This was associated with increased hepatic antioxidant enzymes (SOD). Phenolic compounds in *C. nutans*, such as chlorogenic acid, protocatechuic acid, and caffeic acid, have been shown to reduce oxidative stress markers such as MDA and SOD. Bao et al. (2018) showed that chlorogenic acid decreased MDA and increased SOD levels in the kidney tissue of T2D rats. Likewise, Harini and Pugalendi (2010) demonstrated that protocatechuic acid increased SOD and reduced lipid peroxidation in the plasma, kidney, and liver tissue of T2D rats. Okutan et al. (2005) reported that caffeic acid reduced MDA levels in the cardiac tissue of T2D rat models.

A few mechanisms may contribute to the effects of *C. nutans* in reducing oxidative stress. First, some phenolic compounds in *C. nutans* extract, such as protocatechuic acid, cinnamic acid, and chlorogenic acid, possess antioxidant properties. Free radical species can be neutralized through dismutation or reduction by endogenous antioxidant enzymes, such as SOD (Birben et al. 2012). The high 2,2-diphenyl-1-picrylhydrazyl radical scavenging property in *C. nutans* extract might also increase the presence of antioxidant enzymes and diminish lipid peroxidation in the serum of high-fat- and high-cholesterol-fed rats (Sarega et al. 2016). Second, *C. nutans* may reduce oxidative stress due to CNME improving diabetes-associated hyperglycemia. The hypoglycemic effect of CNME prevents glucose autoxidation, thus reducing the formation of free radical species (particularly superoxide anions). The reduction of superoxide anions increases antioxidant enzyme (SOD) activity, which decreases oxidative stress. Third, *C. nutans* may reduce oxidative stress due to CNME’s ability to improve diabetes-associated dyslipidemia. This property may reduce oxidative stress by mitigating free radicals from the mitochondrial electron transport chain in cellular membranes (Sharma et al. 2012; Sarega et al. 2016). The reduction of free radicals prevents lipid oxidation, in turn reducing MDA levels. By potentiating antioxidant (SOD) status and reducing lipid peroxidation in aortic tissues, CNME attenuates vascular oxidative stress, which has the potential to slow down atherosclerosis development in T2D rats.

Inflammation is a contributor to endothelial dysfunction and plays a significant role in the development of atherosclerosis. At the intima layer of the arterial wall, monocytes release several inflammatory cytokines particularly TNF-α that contribute to vascular endothelium injury and induce vascular wall plaque formation (Sun et al. 2018). The diabetic rats in this study showed increased TNF-α levels, which were reduced after CNME treatment. The effect of CNME in reducing TNF-α levels was comparable with the effects of metformin in diabetic rats. There are currently no *in vivo* studies reporting the effect of *C. nutans* extract on vascular inflammation. However, the current research is in line with a previous *in vitro* study showing reduced inflammatory cytokines, including TNF-α, in lipopolysaccharide-induced inflammation of RAW264.7 cells via *C. nutans* leaf extract (Mai et al. 2016). This effect occurred via inhibition of toll-like receptor-4. It is possible that *C. nutans* reduced inflammatory cytokine TNF-α via substances present in the extract, such as chlorogenic acid and protocatechuic acid. Wu et al. (2014) showed that chlorogenic acid, a substance present in *C. nutans* extract, decreased the serum levels of TNF-α in Apolipoprotein E-deficient mice and inhibited lipopolysaccharide-induced upregulation of TNF-α in RAW264.7 cells. Lin et al. (2009) also demonstrated that protocatechuic acid reduced TNF-α levels in the heart and kidney tissues of diabetic mice. In another study, caffeic acid reduced TNF-α levels in the renal tissue of T2D mice (Chao et al. 2010). In the current study, CNME’s hypoglycemic property might contribute to reducing vascular TNF-α levels.

In the diabetic state, hyperglycemia increases the production of reactive oxygen species, especially superoxide anions. Superoxide anions directly activate transcription factor protein kinase C and nuclear factor kappa-light-chain-enhancer of activated B cells. CNME’s hypolipidemic effect might also contribute to reduced TNF-α levels. In diabetes-associated hyperlipidemia, excess LDL-C leads to an increase in oxLDL-C due to augmented oxidative stress. An increase in oxLDL-C triggers TNF-α gene expression, thus augmenting TNF-α levels (Niemann-Jönsson et al. 2000; Yimin et al. 2012). In this study, reducing LDL-C levels in diabetic rats with CNME, in turn, reduced oxLDL-C formation, which prevented the activation of TNF-α gene expression and thus reduced vascular TNF-α levels.

In this study, the aortic rings of both the DM + CNME and DM + Met groups showed a thinner vascular IMT compared to the untreated diabetic group. Foam cells were seen in the thoracic aortas of the DM group; no foam cells were observed in the CNME- or metformin-treated diabetic groups. CNME’s effect of reducing aortic IMT in diabetic rats was comparable to the results in the metformin-treated diabetic rats. Aortic IMT in the non-diabetic group treated with CNME showed comparable IMT values compared to the non-diabetic control group. A few factors may contribute to the reduction of aortic IMT in diabetic rats treated with CNME. First, it may occur due to CNME’s antioxidant effect. The high 2,2-diphenyl-1-picrylhydrazyl radical scavenging and serum SOD activity of *C. nutans* extract lowers LDL-C oxidation (Katsube et al. 2004; Sarega et al. 2016). Reduced oxLDL-C formation inhibits inflammatory cytokine production, such as TNF-α, which prevents foam cell formation and thus reduces the thickening of the arteries’ intimal layer (Persson et al. 2006; Nguyen et al. 2018). Second, the reduction of aortic IMT through CNME treatment in diabetic rats may result from CNME’s hypoglycemic and hypolipidemic effects, as seen in this study. CNME has previously been shown to increase the expression of the vascular endothelial nitric oxide synthase protein, an enzyme that mediates vascular nitric oxide production (Azemi, Mokhtar, Rasool 2020). This then increases NO bioavailability and prevents leukocyte and platelet attachment to the endothelium to mitigate vascular smooth muscle cell (VSMC) proliferation and migration. The hypoglycemic and hypolipidemic effects also decrease the formation of free radical species but particularly superoxide anions, key activators of VSMC proliferation. Reduced superoxide anion formation can prevent the secretion of cyclophilin A (CyPA). CyPA is a potent chemoattractant for monocytes that elicits an inflammatory response (Jin et al. 2000). Reduced CyPA reduces vascular adhesion molecule-1 and E-selectin expression, thus reducing VSMC proliferation and intimal layer thickening. Several *in vitro* studies have demonstrated that protocatechuic acid, caffeic acid, gallic acid, and luteolin, substances present in *C. nutans*, inhibit VSMC proliferation (Li et al. 2005; Lin et al. 2015; Wu et al. 2018; Chung et al. 2020). Chen et al. (2009) also showed that lithospermic acid, a caffeic acid derivative, inhibits VSMC proliferation and migration, which leads to therapeutic effects against neointimal hyperplasia and atherosclerosis.

## Conclusions

The 4-week treatment of diabetic rats with CNME reduced serum TC, TG, LDL-C, and AI levels; improved vascular antioxidant capacity and oxidative damage; and reduced vascular inflammation and aortic IMT. These effects were comparable to metformin and suggest that CNME has the potential to reduce atherosclerotic risk in T2D rats.
